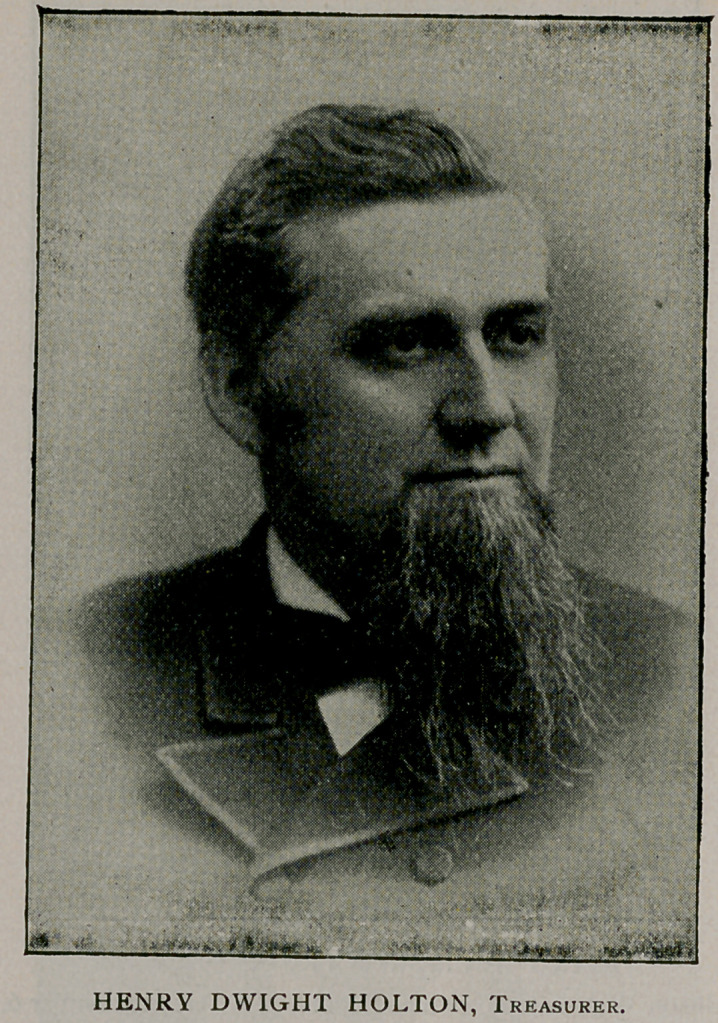# American Public Health Association: A Historical Sketch—History of the Preparation for the Buffalo Meeting, 18961In the preparation of this article I have drawn liberally on information furnished by the secretary of the association, Dr. Irving A. Watson, of Concord, N. H.

**Published:** 1896-09

**Authors:** William Warren Potter

**Affiliations:** Buffalo, N. Y.


### Special Article.

### AMERICAN PUBLIC HEALTH ASSOCIATION: A HISTORICAL SKETCH—HISTORY OF THE PREPARATION FOR THE BUFFALO MEETING, 1896.[1]

By WILLIAM WARREN POTTER, M. D., Buffalo, N. Y.

PROBLEMS relating to preventive medicine, and especially those connected with public sanitation, are presenting themselves for solution in constantly increasing numbers and with more forceful accentuation as time advances. Methods and habits of life are constantly changing, causes of disease that existed a century ago are disappearing, while new ones are presenting themselves with a frequency that almost rivals the phases of the moon.

Just as the nineteenth century is drawing near its end, the time seems appropriate for the consideration of many of these interesting problems, hence Buffalo may consider it a fortunate circumstance that the American Public Health Association has appointed its annual meeting for 1896 to be held in this city. This circumstance makes the occasion for presenting the purposes of this organization in considerable detail an appropriate one, and

1. In the preparation of this article I have drawn liberally on information furnished by the secretary of the association, Dr. Irving A. Watson, of Concord, N. H.

what is here offered may assume, for that reason, the form of a historical sketch.

The American Public Health Association was organised in 1872 by a few public-spirited men, who foresaw the propriety of grouping together in one society some of the ablest sanitarians in the country, whose province it should be to inaugurate measures for the restriction and prevention of contagious and infectious diseases, and for the diffusion of a knowledge of sanitary science among the people.

It is interesting to note what remarkable growth this association has developed; for, whereas in 1872 it comprised only a little group of sanitarians who met together in the parlor of a hotel for conference, we find it in 1896 to be the largest public health association in the world, embracing in its jurisdiction the three great countries of the North American continent,—namely, the United States of America, the Dominion of Canada and the Republic of Mexico, representing in the aggregate a population of 85,000,000 of people.

Let us pause a moment and glance at its first meeting, that it may be contrasted at a later period with the one about to be held in Buffalo. Upon invitation a few gentlemen interested in the study of sanitary science met at the New York hotel, in the City of New York, on Friday evening, April 18, 1872, with the avowed purpose of creating a permanent association for promoting public health interests. Those present on this occasion were: Drs. Stephen Smith, Elisha Harris, E. H. Janes, Heber Smith and Moreau Morris, of New York; Dr. Francis Bacon, of New Haven; Dr. Christopher C. Cox, of Washington; Dr. John H. Rauch, of Chicago, and Carl Pfeiffer, Esq., of New York City.

At this meeting it was decided to form a permanent organisation, and the names of several others were added to the list as founders.

The second meeting was held at the Ocean hotel, Long Branch, N. J., September 12, 1872. On this occasion, according to the records, only twelve persons were in attendance. However, a proposed plan of organisation was presented, which afterward was revised and adopted as the constitution of the American Public Health Association. At this session seventy persons, nearly all of whom were physicians interested in sanitary work, were elected to membership. Sanitary topics were discussed, committees appointed, officers chosen, the organisation perfected in considerable detail and the meeting adjourned amidst commendable enthusiasm.

The next meeting was held at Cincinnati, May 1, 2 and 3, 1873, and this, for the first time, was dignified with the name of the "annual meeting." The association by this time had begun to manifest considerable activity, and though the records show that but seventeen persons were present at this session, there were several valuable papers read and discussed, and important resolutions were adopted relating to public health topics.

The next annual meeting was held in New York City, November 11, 12, 13 and 14, 1873. Here again several papers relating

HOTEL NIAGARA.

to sanitary science were read, and on the second day of the meeting the following preamble and resolution were adopted:

WHEREAS, The roll of members being called, it was found that the constitutional quorum of twenty-five was not present, and the same difficulty having occurred at all the previous meetings since the organisation of the association, on motion of Dr. J. J. Woodward, U. S. Army, the association was dissolved and adjourned *sine die*. It was then,

*Resolved*, That the members of the late association present proceed to form an organisation to be called the American Public Health Association.

The original constitution was then amended in several particulars, the most important of which was to make nine members constitute a quorum for the transaction of business. It was also voted that the association assume all pecuniary liabilities of the late American Public Health Association.

Therefore, according to record, the American Public Health Association, as it now exists, was legally organised November 12, 1873, although it was in fact created April 18, 1872, as before stated.

Since the time of its preliminary meeting at New York, in 1872, it has met in many of the principal cities of the Union as well as in the Dominion of Canada, and it has held one meeting in the City of Mexico.

In twelve years from its initial meeting the association had become so strong and influential that at the St. Louis meeting, in 1884, a proposition was made to include Canada within the territorial area of its jurisdiction, and a change in the constitution to accomplish this end was proposed. It lay on the table for one year and was adopted at the Washington meeting, in 1885, thus giving representation to the Dominion of Canada and its several provinces in the association.

Later it was proposed to interest the Republic of Mexico in the work of the association, and with that end in view, in 1890, the secretary, Dr. Irving A. Watson, of Concord, N. H., was instructed to invite the Government of Mexico and the National Board of Health of that country to send delegates to the Charleston meeting. The invitation was accepted and two delegates from Mexico were present at Charleston, December 16–19, 1890. In 1891 the meeting was held at Kansas City, when a proposition to amend the constitution, so as to admit Mexico and its several states, was presented and it was adopted at the meeting held in the City of Mexico, in 1892.

With great appropriateness this year the president, Dr. Eduardo Liceaga, is a Mexican, and will attend the Buffalo meeting with a number of accredited delegates from the republic of Mexico.

To state the matter in brief, it will be observed from the foregoing résumé that the association has grown in twenty-four years from a small coterie of sanitarians, to be the largest health organisation in the world, embracing in its territorial jurisdiction three great countries, which comprise nearly all of North America.

During this time the association has issued twenty large volumes of annual reports, which contain the papers read and discussions held at its annual meetings; besides, it published in a separate volume the work of one of its committees, entitled Disinfection and Disinfectants, which contains a large amount of original research, and which has taken its place as authority in the literature of the subject. Moreover, it has published the Lomb Prize Essay, over 100,000 copies of which have been distributed. Many of these papers and reports have also been widely circulated in pamphlet form. Since 1894 the association has published its transactions in the form of a quarterly journal, which may be bound uniformly with the preceding volumes, and which is one of the handsomest publications that the JOURNAL receives in exchange. The publications of the association alone constitute a valuable library upon sanitation, and may be considered as authoritative in regard to

ELLICOTT SQUARE BUILDING.

public health questions. They are well printed and beautifully bound, and are alone worth more to those interested in the subject with which they deal than is the cost of membership in the association.

It is supposed by many that the association's membership is made up entirely of physicians. This, however, is not the case. On its rolls may be found not only the names of physicians, but also of lawyers, ministers, civil and sanitary engineers, health officers, teachers, plumbers, merchants, business men of various sorts, and indeed almost every profession as well as industrial pursuit is represented in its list of members.

The record of the association from its organisation to the present time has been one of steady and constant progress, while its influence on sanitary legislation and in public health affairs is beyond limit. It was never so strong as now, and the high character of the organisation makes it desirable to obtain membership in it.

Turning now to the prospective Buffalo meeting, let us say a few words. Last year the association met at Denver, and Dr. Ernest Wende, health commissioner of Buffalo, went as a delegate, authorised by the mayor and the common council to invite the association to hold its twenty-fourth annual meeting in this city. Dr. Wende was successful in obtaining an acceptance of the invitation, and now is most active in endeavoring to fulfill his promise to the association—namely, that it should be adequately and generously entertained during its visit to this city. At Denver he was appointed by the association chairman of the local committee of arrangements for this year, and acting on that authority has in turn appointed a number of committees, each member of which may be said to be, constructively speaking, one of his official staff.

The organisation is as follows: Chairman of local committee of arrangements, Dr. Ernest Wende; of finance committee, Dr. Henry Reed Hopkins; of committee on membership, Dr. Walter D. Greene; on transportation, Mr. Edson J. Weeks; of reception and entertainment, Dr. Lucien Howe; of printing and badges, Dr. William Warren Potter; on halls and meeting places, Dr. Wm. C. Phelps; on hotels, Dr. J. B. Coakley; on exhibits, Dr. Byron H. Daggett; of information, Dr. Eugene A. Smith; executive committee, Dr. E. Wende, chairman; Mr. J. D. Wood, secretary.

Each of these chairmen has selected a number of gentlemen to act with him, who are especially interested in the success of the meeting. The executive committee, consisting of the chairmen of the several committees together with other appointees, meets every Friday, at 4 o'clock, at the Ellicott Club, for conference and discussion of ways and means through which to perfect the organisation and bring success to the enterprise.

To those unaccustomed to deal with these questions it would seem, perhaps, an easy matter to prepare for such a meeting. Such, however, is not the case. The details are manifold, and some of them embrace problems of intricacy and business importance. It requires vigilance, activity and perseverance on the part of the committees to obtain success in such an undertaking, and, moreover, harmony of action and concentration of authority are essential. These happily have been obtained through the selection of the chairman of the executive committee, a man of affairs who brings himself in touch with the chairmen of the other committees almost daily, and surely as often as once a week. The question of greatest importance in regard to entertaining such an association is finance. Without an ample supply of money little can be accomplished that will prove satisfactory, either to the citizens or to the association. This part of the work, fortunately, has been placed in the hands of Dr. Henry Reed Hopkins, who is progressing most satisfactorily. There is yet, however, great need of contribu-

BUFFALO LIBRARY.

tions, and it is to be hoped that our business fellow-citizens will respond to the appeals sent out for aid in behalf of the project, with a liberality characteristic of the men who are asked to give.

Nothing contributes to business success more than good health, either individual or public. To keep a large city healthful costs money, but it is an investment that pays very large dividends. The cost of sickness is indeed one of the most expensive items that becomes a charge on the private purse. So, too, an epidemic of disease, contagious or infectious, is an expensive luxury for any city to indulge in. In this age of the world there is scarcely an epidemic disease known that is not preventable, and it is the duty of the citizens to provide the health department with sufficient means to prevent or control such epidemics. There must be just such meetings as the one contemplated in Buffalo, of scientific men to discuss these problems with reasonable frequency, in order that the best methods may be made known to our local health officers. Every one of our business men is interested in this question, and ought to feel a pride in the entertainment of the association on lines commensurate with the dignity and standing both of the city and the visitors. No doubt they will when the importance of the subject is presented in a proper light.

The committee has selected Ellicott Square in which to hold the meetings of the association, where ample accommodations are found, both for the general sessions and for the transaction of business connected with the association. There are adequate committee rooms, assembly rooms, offices and exhibition halls, and altogether the place chosen will be found not only useful but attractive. Beside the regular scientific sessions of the association there will be provided certain social entertainments of an agreeable nature. Among the latter may be mentioned a reception to be given at the Ellicott Club during one of the evenings, an excursion to Niagara Falls, going down to Queenstown on the Canadian side, and returning by the Gorge road on the American side, and a collation provided at Niagara Falls. This will be one of the most attractive and systematically arranged excursions that has ever been given over the route named. The excursionists will return to Buffalo by the Niagara Electric Railway. In addition to these, there will be provided steamboat and tally-ho rides, as well as other incidental ways of entertainment, none of which, however, will interfere with the regular sessions.

While the business office of the association will be located in Ellicott Square, the Niagara hotel has been chosen as the general hotel headquarters for the visitors. It is well adapted to this purpose, both in its environment and management. It is a little removed from the noisy centers, is easily accessible, and when reached affords all the comforts of a metropolitan hotel, ensconced in almost rural surroundings.

It is desirable that the medical profession of Buffalo and vicinity, together with such other professional and business men as feel interested in the subject, shall make early application for membership. This is one way to manifest interest in the association and an important method of contributing to the success of the Buffalo meeting. The necessary requisites are good professional and moral standing, an interest in hygiene, the endorsement of two members of the association and the payment of $5, which is the regular membership fee. The transactions, when published, will be more valuable than the fee for membership.

We bespeak from the friends of public health, hygiene and general preventive medicine everywhere an earnest coöperation

VIEW ON DELAWARE AVENUE.

with the committees headed by Dr. Ernest Wende, in support of the general scheme outlined above, that has for its object the proper entertainment of a learned body of men, who are engaged in a humane work and who deserve most generous treatment at the hands of every public-spirited person in the city of Buffalo.

We conclude this subject by presenting portraits and biographical sketches of the officers of the association who will conduct the Buffalo meeting.

The half-tones are from "Physicians and Surgeons of America," by Irving A. Watson, M. D., Concord, N. H., from which source also the biographies were abstracted.

It is to be hoped that what has been said in this paper will stimulate our readers to an interest in the association, that will be manifested through an attendance at the meeting.

EDUARDO LICÉAGA, President.

Eduardo Licéaga was born October 13, 1839, at Guanajuato, Mexico. He was educated at San Gregorio National College in the City of Mexico, and received several prizes during his collegiate training. He was five years a student at the National College of Medicine, City of Mexico, and received his degree of M. D. January, 1866, and from the Emperor Maximilian a gold medal for having secured the premiums in all the professional courses. He commenced the practice of his profession soon afterward in the City of Mexico, and has remained there ever since. Dr. Licéaga is corresponding member of the Society of Medicine, San Luis Potosi, 1872; titular member of the National Academy of Medicine, 1873, having filled the offices of president and vice-president; member of the Pharmaceutical Medical Society, Puebla, 1874; fellow-laborer member of "Larrey" Medical Surgical Association, 1875; honorary member of the "Sociedad Filoiatrica y de Beneficencia" of the National School of Medicine, 1878; member of the Medical Society of Guanajuato, 1879; corresponding member of the Public Hygiene Society of Paris; is permanent president of the Superior Board of Health of Mexico; member of the American Public Health Association, vice-president, 1893; prefect of the National School of Medicine, 1867–70, and director *pro tempore;* president of the Mexican Medical Congress of Hygiene, 1876–78; director of Maternity and Infants' Hospital, City of Mexico; professor of surgical therapeutics, National School of Medicine; secretary, treasurer and vice-president successively of the Sociedad Filharmonica; professor of acoustics and phonography in the Musical Conservatory, 1868–72; member of the Compañia Lancasteriana; member of the Mexican Society of Geography and Estadistics; and is secretary-general of the Second Pan-American Medical Congress to be held in the City of Mexico, November 16, 17, 18 and 19, 1896. Dr. Licéaga is believed to have been the first in the Republic of Mexico to perform with success coxo-femoral resection in a child, obtaining reproduction of the bone and recovery of the functions of the member. He is the author of many valuable medical, surgical and other monographs. He was married December 24, 1870, to Miss Dolores Fernandes de Zanregni, and has four children. At the last meeting of the American Public Health Association, held in October, 1895, at Denver, Dr. Licéaga was elected president, and will officiate at the 24th Annual Meeting in Buffalo, September 15–18, 1896.

ALFRED ALEXANDER WOODHULL, VICE-PRESIDENT.

Alfred Alexander Woodhull was born April 13, 1837, at Princeton, N. J. His father was a physician and his paternal grandfather a clergyman. He graduated from Princeton College, receiving the degrees A. B. 1858, A. M. 1859, and LL. D. 1894. He received the degree of M. D. from the University of Pennsylvania 1859. He first practised his profession at Leavenworth, Kans., and then at Eudora in the same state. In the spring of 1861 he helped to raise a company of mounted rifles in which he was commissioned a lieutenant, but in September, 1861, he was appointed an assistant surgeon in the U. S. army. He served during the war in the field with troops, and as assistant to medical directors, and was acting Medical Inspector of the Army of the James in 1864–65. In March, 1865, was brevetted lieutenant-colonel and attained the actual rank May 16, 1894. Colonel Woodhull is a member of the Association of Military Surgeons of the United States; of the American Public Health association; and a companion of the military order of the Loyal Legion of the United States. He was appointed Medical Director Department of Colorado, July 16, 1895, with headquarters at Denver. He represented the medical department of the United States Army at the Eighth International Congress of Hygiene and Demography, London, 1891; was instructor in military hygiene at the Infantry and Cavalry School, Fort Leavenworth, 1886–90; and was in command of the Army and Navy General Hospital, Hot Springs, Ark., 1892–95. His literary contributions have been numerous and valuable, and he is a gold medalist of the Military Zouave Institution for a prize essay on "The Enlisted Soldier," published in its Journal March, 1887. He married, December 15, 1868, Margaret, daughter of Elias Ellicott of Baltimore. At the meeting in Denver, Col. Woodhull was elected vice-president of the American Public Health Association, and will take an active part in the Buffalo meeting, September 15–18, 1896.

HENRY SEWALL, Vice-President.

Henry Sewall of Denver, Colorado, received his preliminary education at the Wesleyan University, Middletown, Conn., from which he obtained the degree of B. S. in 1876, and at Johns Hopkins University, Baltimore, which granted him the degree of Ph. D. in 1879. He received his medical education at the University of Michigan, taking the doctorate degree in 1888. The Medical Department of the University of Denver conferred upon him the degree of M. D. *adeundem* in 1889. He is professor of physiology in the University of Denver; Assistant Health Commissioner of Denver, and secretary of the Colorado state board of health. He is a member of the American Association of Physicians; of the Colorado State Medical Society; of the Denver Medical Association; and of the Araphoe County Medical Society. In 1895, Dr. Sewall was elected vice-president of the American Public Health Association and will give active support to the Buffalo meeting.

IRVING ALLISON WATSON, SECRETARY.

Irving Allison Watson was born at Salisbury, N. H., September 6, 1849. He received his preliminary education in the common schools of New Hampshire, and at the Newbury (Vt.) seminary and collegiate institute. His medical education was obtained at Dartmouth Medical College and at the University of Vermont, from which latter he took his doctorate degree in 1871. In 1885 Dartmouth College conferred upon him the degree of A. M. Dr. Watson began the practice of his profession at Groveton, Vt., where he resided for ten years. During this time he served for a number of years as Superintendent of Schools, and was twice elected to the legislature. He was instrumental in securing the passage of the law creating the state board of health, and was chosen secretary and executive officer upon its organization, September, 1881. He removed to Concord in the latter year, and was made executive officer of the state board of lunacy, when the latter board was organized in 1889. Dr. Watson is registrar of vital statistics of New Hampshire; president of the state board of cattle commissions; and has been secretary of the American Public Health Association since 1883; was vice-president of the International Conference of State Boards of Health, 1894; is a member of the American Medical Association; is an honorary member of the Academia Nacional de Medicina de Mexico; was assistant secretary-general of the First Pan-American Medical Congress; is a member of the Société Française d'Hygiène; of the medico-legal society of New York; of the New Hampshire Medical Society, and of his local medical societies. He is also a registered pharmacist of the State of New Hampshire. Dr. Watson has compiled and edited the New Hampshire Registration Reports since 1881; the reports of the state board of health, thirteen volumes; the transacts and journal of the American Public Health Association since 1883; and the Reports of the Commissions of Lunacy of New Hampshire, five volumes. He has also been a frequent contributor to the medical periodical press, especially on the subjects of public sanitation and hygiene. He married, in 1872, Lena A., daughter of Gilman Farr of Littleton, N. H., and has one child, a daughter, Bertha M. Watson. Dr. Watson is the executive officer of the American Public Health Association, and will take up his headquarters in Buffalo several days before the meeting, to assume the administration of its details.

HENRY DWIGHT HOLTON, TREASURER.

Henry Dwight Holton of Brattleboro, Vt., was born at Rockingham, Vt., July 24, 1838, and was educated at Saxton's River (Vt.) Academy. He was a pupil of Dr. J. H. Warren of Boston, and Dr. Valentine Mott of New York, and graduated from the University of New York in 1860. He practised first in Brooklyn, then at Putney, Vt., and has lived in Brattleboro twenty-seven years. He is a member of the Connecticut River Medical Society, of the Vermont Medical Society, of the American Medical Association, of the British Medical Association, of the American Public Health Association, and of the Gynecological Society of Boston. He has been president of local medical societies, of the Vermont Medical Society, and vice-president of the American Medical Association. He is the author of several important medical papers that have been published in society transactions and medical journals. He was for 14 years professor of therapeutics and general pathology in the medical department of the University of Vermont In 1884 he was elected to the state senate, and in 1888 he represented his town in the Vermont legislature. He has been three times elected by the legislature trustee of the University of Vermont, the term of office being six years ; was appointed by the Governor as Commissioner to represent the State in the National Nicaragua Canal Convention ; also a Commissioner for the Columbian Exposition. He was president of the board of trustees of the First Pan-American Medical Congress, and has held several local public offices. He married, November 9, 1862, Ellen J. Holt. Dr. Holton was elected treasurer of the American Public Health Association at its meeting in the City of Mexico, December, 1892, and has held the office since that date. He will take an active part at the Buffalo meeting.

## Figures and Tables

**Figure f1:**
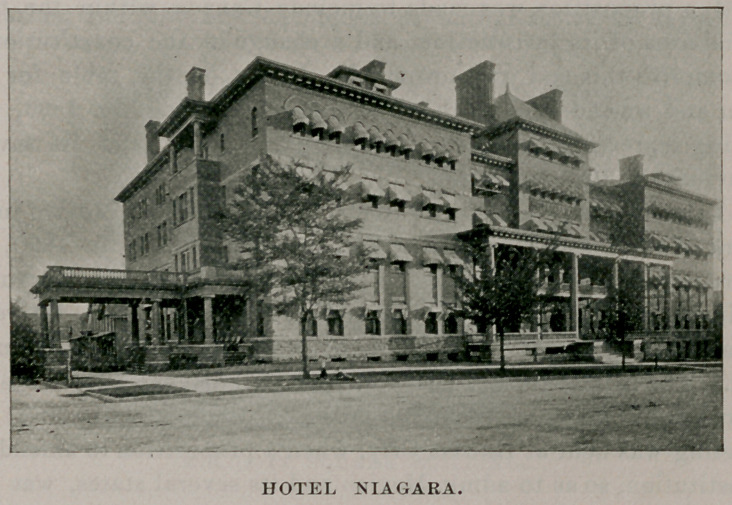


**Figure f2:**
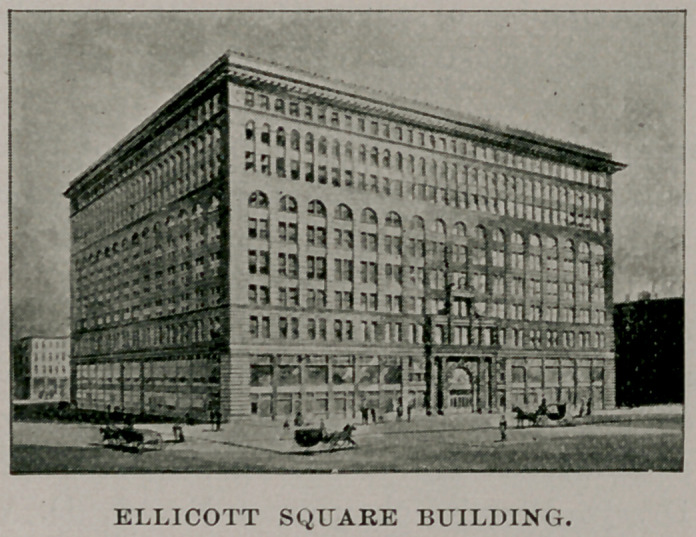


**Figure f3:**
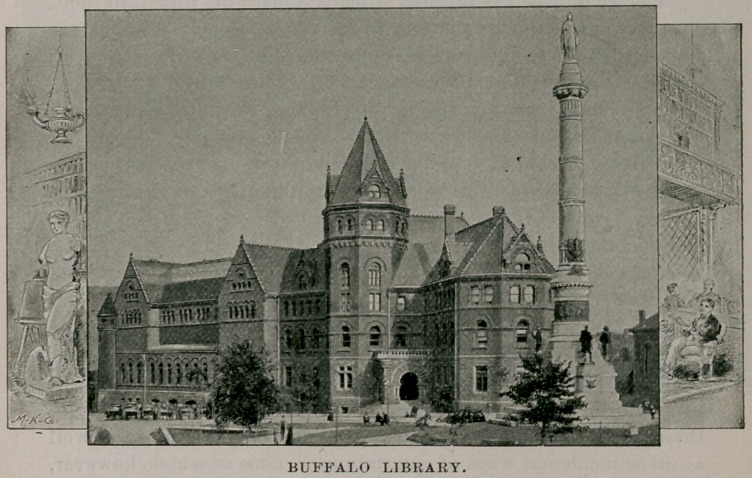


**Figure f4:**
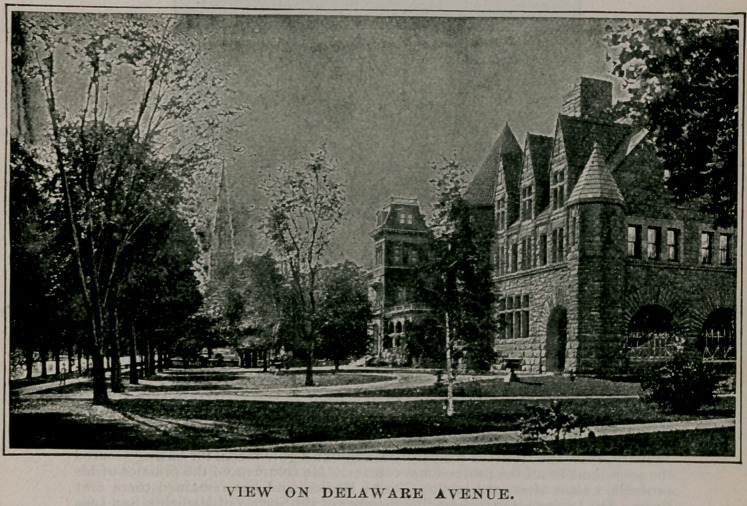


**Figure f5:**
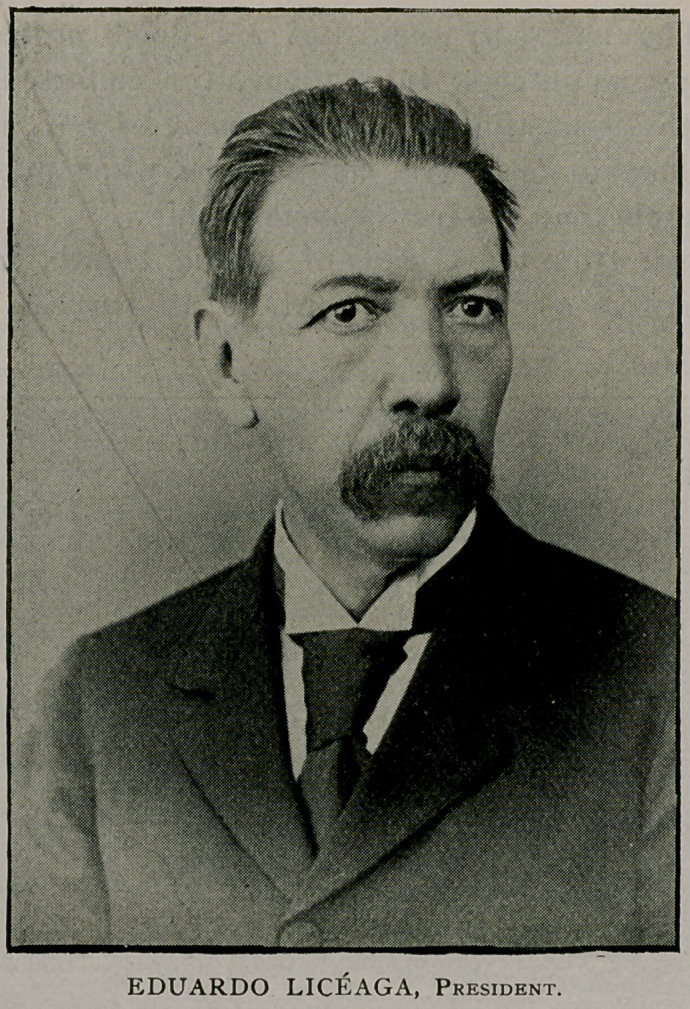


**Figure f6:**
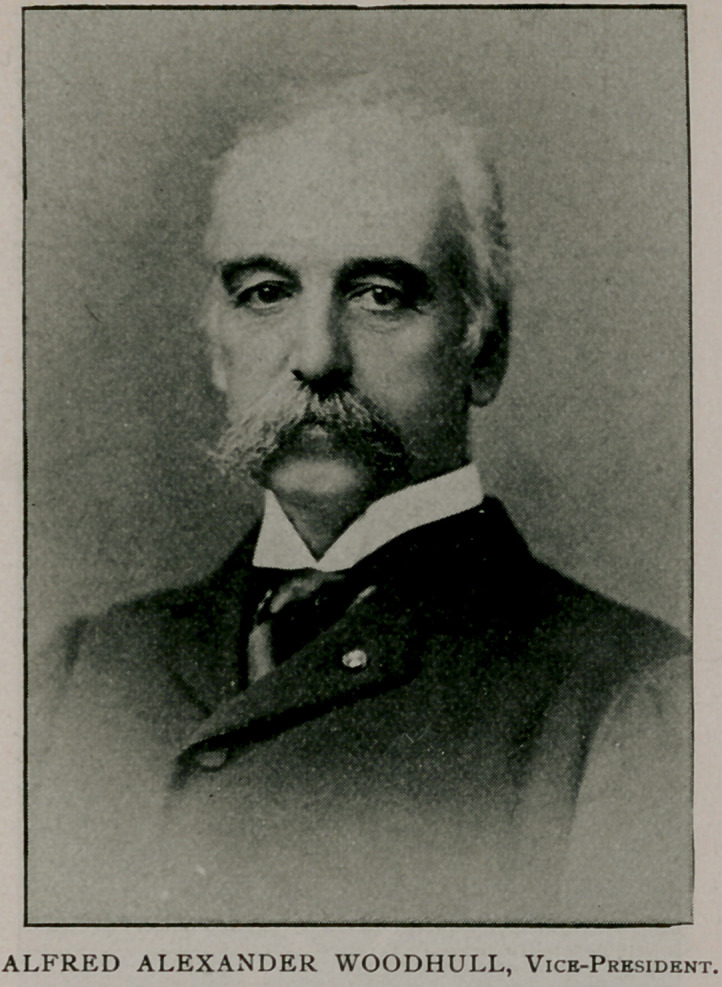


**Figure f7:**
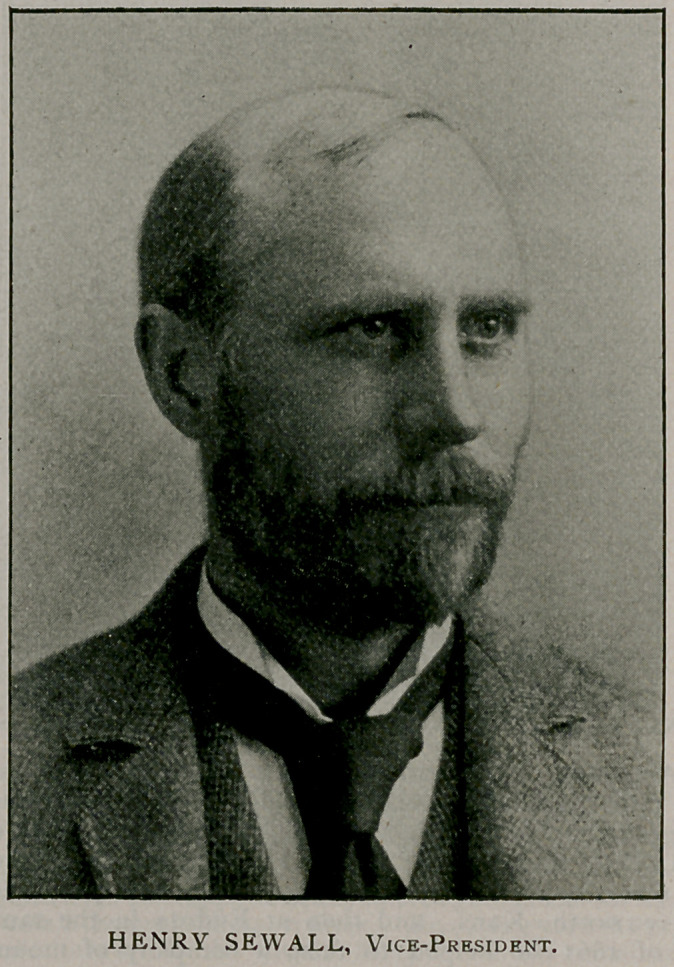


**Figure f8:**
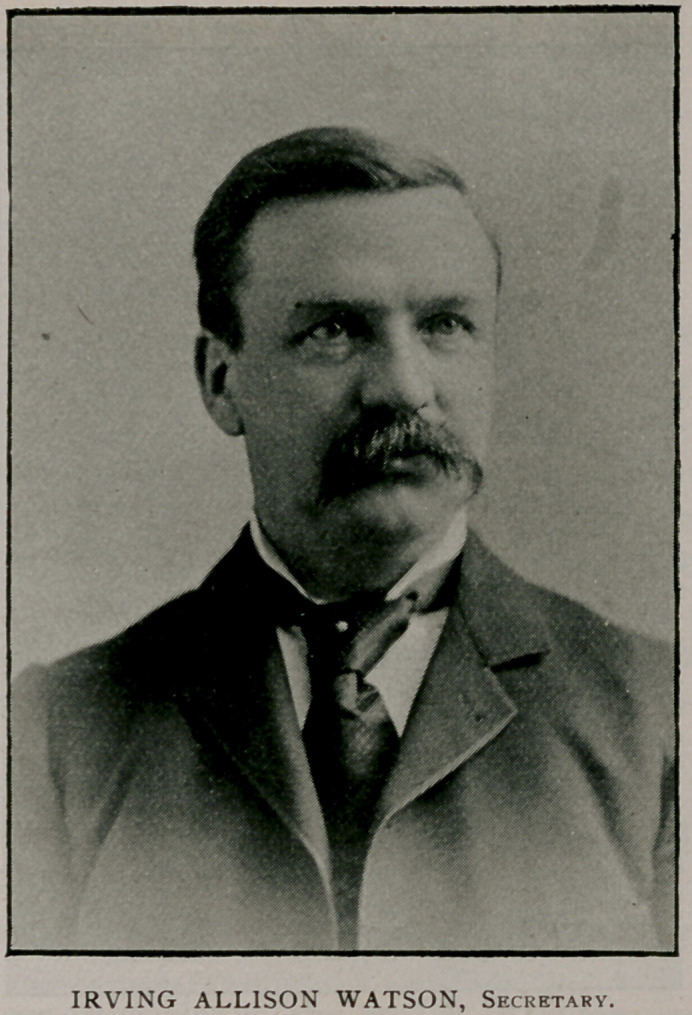


**Figure f9:**